# Phytochemical profiling and anthelmintic potential of extracts of selected tropical plants on parasites of fishes in Epe Lagoon

**DOI:** 10.1038/s41598-023-48164-8

**Published:** 2023-12-20

**Authors:** U. D. Ukwa, J. K. Saliu, B. Akinsanya

**Affiliations:** 1 Department of Zoology, Eco-Toxicology Unit, University of Lagos, Akoka, Lagos, Nigeria; 2 Department of Zoology, Environmental Parasitology Unit, University of Lagos, Akoka, Lagos, Nigeria

**Keywords:** Plant sciences, Zoology, Ecology, Diseases

## Abstract

This research aims to study the anthelmintic properties of selected five (5) tropical plant extracts, ascertained margin of fish host safety in reference with praziquantel, a commonly used chemo-therapeutics. Qualitative and quantitative analysis of Alligator pepper seeds (*Aframomum melegueta*), Moringa leaves (*Moringa oleifera*), Neem leaves (*Azadirachta indica*), Ginger bulbs (*Zingiber officinale*) and Garlic (*Allium sativum*) and their potencies in reference to praziquantel against *Clarias gariepinus* and different classes of helminth parasites were investigated. The results obtained show that the 70% ethanol extract had 80 to 100% presence of the phytochemical content, compared with the 100% aqueous and 100% ethanol extracts with 50 to 80% and 50 to 90%, respectively. Among the five tropical plants, the richest in saponin and flavonoids are alligator pepper and neem with alkaloids, tannin, flavonoid and saponin in ratios 1:1:3:9 and 1:1:4:3 respectively. While, moringa, garlic and ginger are rich in alkaloids with alkaloids, tannin, flavonoid and saponin in ratios, 8:1:10:1, 6:2:1:4 and 6:3:2:1, respectively. *Aframomum melegueta* and praziquantel showed above 70% potency (at 96 h LC_5_) against all the classes of parasites; W*enyonia spp* (cestode), *Procamallanus spp* (nematode), *Tenuisentis spp* (acanthocephalan), and *Electrotaenia sp* (cestode) as compared to the other plant extracts that showed above 70% potency (at 96 h LC_5_) only against *Electrotaenia spp.* Sub-lethal Concentrations (96 h LC_5_) of praziquantel and *Aframomum melegueta* on the juvenile fish host (12.36 mg/l and 9.9 mg/l respectively) were found to be 90.9% and 93.5% effective against adult *Electrotaenia spp* after 8 to 10 min of exposure. These concentrations were 78 to 85.7% and 89.7 to 88.4%, respectively, effective against the other classes of parasites after 18 to 25 min and 15 to 21 min of exposure. These concentrations were tested on the post juvenile of the fish to determine behavioral changes; there were no significant behavioral responses after 24 h of exposure. The effective concentrations indicate the widest margin of safety for the fish host.

## Introduction

Nigeria's Aquaculture industry is one of the most rapidly expanding food-producing sectors in the country, the third largest in Africa. The industry had 12.3% annual growth between 2000 and 2020, higher than regional and world averages (FAO, 2022). In spite of this, the demand for fish is high due to the increasing population. The decline in fish productivity is attributed to parasitic diseases (Foysal et al., 2019). Common parasitic diseases of cultured fishes are caused by both ecto and endo parasites belonging to the groups; protozoa, trematodes, nematodes and cestodes. Some fish parasites have been reported to cause diseases in humans, like Anisakiasis and Diphyllobothriasis caused by fish nematode and cestode parasites^1^, Lima dos Santos and Howgate, 2011). *Procamallanus spp*, *Gyrodactylus spp*, *Cleidodiscus spp*, *Orientacreadium spp* and *Polyonchabathrium spp* and *Icthyophthirius spp* are commonly reported parasites in the cultured system (Abdel-Gaber et al., 2015;^2^). *Clarias gariepinus* has been reported to show high parasitemia of both medical and economic importance^3–5^.

Chemotherapeutants have been commonly and effectively used to control and treatment of fish parasites. These anthelmintics have their demerits. Some of them have broad-spectrum anthelmintic activity with a narrow margin of safety, considering the significant effects on the host and the environment (Palma et al., 2015). These drugs enter the environment through treatment processes, improper disposal of used containers and waste waters, unused drugs or fish feed, causing environmental pollution. Another major demerit is the accumulation of chemical residues in fish tissues and parasite resistance. Some chemotherapeutic residues have been reported in tissues of cultured fish^6^, Jung et al., 2021). Praziquantel, highly recommended and widely used, has numerous advantages like the development of resistance^7,7^] , being hazardous for animal health and environmental disadvantages.

Medicinal plants have been reported as promising sources of therapeutic agents for the treatment and control of parasitic diseases in fish^9–13^, Doligalska et al., 2021). Previous studies on extracts of tropical plants indicated that *Afranamum melegueta* and *Azadirachta indica* show higher therapeutic potential against helminth parasites; *Wenyonia spp* and *Procamallanus spp* compared to other plants such as *Piper guineense*, *Moringa oleifera*, *Gongronema latifolium*, *Garcinia kola* and *Xylopia aethiopica*^9,10^. The unique phytochemical property of these plants is, they are rich in saponins. The ratio of saponins, alkaloids and flavonoids present in *Afranamum melegueta* and *Azadirachta indica* is (8:2:1) and (4:1:1) respectively. Saponins eradicated *Heligosomoides polygyrus bakeri,* a nematode larva L3 by altering the subcellular morphology and preventing nematode-specific proteins (Doligalska et al., 2021). Michela et al., (2020) reported inhibition of egg production in species of gastrointestinal strongyle nematodes (GIS) by saponins from *Medicago spp* in a concentration-dependent manner compared to the reference drug, thiabendazole.

The objective of this study was to determine anthelmintic properties of selected five (5) tropical plant extracts and ascertained the margin of fish host safety in reference to a commonly used chemotherapeutic drug.

## Materials and methods

### Plant materials

Selected five (5) tropical plants; Alligator pepper seeds (*Afranamum melegueta*), Moringa leaves (*Moringa oleifera*), Neem leaves (*Azadirachta indica*), Ginger bulbs (*Zingiber officinale*) and Garlic (*Allium sativum*) were purchased from Iyana-Ipaja market, Lagos State. The plant materials were authenticated in the Department of Botany, University of Lagos, Akoka, Nigeria.

### Preparation and extraction of plant materials

The fresh plant parts were washed and air-dried for a period of one month. They were pulverised using an electric grinder. Extraction was done using the solvent extraction method (maceration). Solvent extracts used include; 100% ethanol (undiluted), 70% ethanol (diluted) and distilled water. Hundred grams of powered samples were weighed and soaked in a stopped container with 250 ml of the solvent for 72 h and rapidly stirred using glass rod every 6 h. After 72 h, filtration was concentrated using a vacuum drying oven for 72 h. The extracts were refrigerated at 4ºC until required for the assay.

### Phytochemical analysis

#### Qualitative analysis

The extracts were screened for the presence of active constituents with the aid of respective solvents as described by Sofowora^14^. Alkaloids, Flavonoids, Saponins, Tannins, Terpenoids, Cardioglycosides, Phlobatannins and Steriods were screened.

#### Quantitative analysis

Alkaloids, flavonoids, saponins and tannins were quantified using the methods described by Harbone, (1973), Bohman and Kocipai (1974), Obadoni and Ochuko^15^, ^16^ respectively.

#### Chemo-Anhelmintics

6oomg of praziquantel tablets were obtained from the HealthPlus Pharmacy in Sabo Yaba, Lagos State. These tablets were grinded to powdery form and dissolved in 96% ethanol to prepare the stock solution. This is due to low solubility in water. The praziquantel dissolved in ethanol was equivalent to concentrations; 20, 30, 40, 50, 60 and 70 mg/l.

#### Fish model – Clarias gariepinus

One thousand samples of two weeks old fingerlings of *Clarias gariepinus* with weight ranged, 1.25 g to 2.03 g and one hundred juveniles with weight ranged, 18.6 g to 23.6 g were bought from a local Aquaculture Unit located in Egbeda, Lagos State Nigeria. The specimens were acclimatized to the laboratory condition for a period of 14 days.

#### Acute toxicity test

96 h LC_50_ value of the five plant extracts and praziquantel were determined using fingerlings and were conducted in static system in accordance to the OECD guideline^17^. Each extract was added to one litre of distilled water to prepare the stock solution. The range finding test was determined according to the method described by Solshe and De (1993) using five fishes as test animals. Gentle aeration of test vessels in a static renewal in five test concentrations was employed. Mortality observations were recorded every 24 h,24 h, 48 h, 72 h and 96 h.. They were taken to be dead if they show no body movement even when probed with a rod and appear whitish.

#### Toxicity factor

Relative Toxicity Factor (RTF) for each plant extracts and praziquantel at 96 h of lethal exposure were estimated using the formulae below modified from Akinsanya et al.^10^,

$${\text{Relative Toxicity Factor }}\left( {{\text{RTF}}} \right) \, = \frac{{{\text{96 h LC}}_{{{5}0}} {\text{value for each extract and praziquantel}}}}{{{\text{Plant extract and chemo}} - {\text{anthelmintics with least 96 h LC}}_{{{5}0}} }}$$

#### 24 hour behaviourial assessment of post juvenile of Clarias gariepinus

8 weeks old juveniles of *Clarias gariepinus* with mean weight of 22.6 g were assessed for behavioural responses after 14 days acclimatization. They were exposed to sub-lethal concentrations (96 h LC_5_, 96 h LC_10_) and lethal concentrations (96 h LC_50_, 96 h LC_95_) of each of the five plant extracts and praziquantel for 24 h. The specimens were assessed for rapid movement, faster opercular activity, erratic swimming, and loss of balance, respiratory distress, lethargy and respiratory failure.

#### Assessment of helminth parasites in freshwater fishes in Epe lagoon

Three hundred and sixty (360) post juvenile samples of ray-finned fishes comprising of *Clarias gariepinus, Synodontis clarias, Heterotis niloticus* and *Malapterurus electricus* were caught from the Epe lagoon, Lagos State in wet season between June and September, 2022 and dry season between December and March, 2023. The gastrointestinal tract of individual fish was cut open and examined for the presence of parasites. The recovered worms were placed in a petri dish containing 0.85% saline and used for the toxicological assessment.

#### In-vitro exposure of parasites to the plant extracts and chemo-anthelmintics

An in-vitro study was conducted using sub-lethal concentrations (96 h LC_5_, 96 h LC_10_) and lethal concentrations (96hLC_50_, 96 h LC_95_) of each of the five plant extracts and praziquantel helminth parasites of fish. Saline solution was used as the control. There were two replicates (each containing three parasites) for each treatment concentration. The worms were immediately put into different petri dishes containing the different extracts and chemo-anthelmintics and timed to determine the Average Survival Time (AST). AST for the various classes of the parasites (cestodes, trematodes, acanthocephalans and nematodes) in saline solution (0.0085 g/ml) were estimated. Percentage Reduction in Survival Time (%RST) was estimated using the formulae below;

$$\% {\text{ Reduction in Survival Time}} = \left( {\frac{{{1} - {\text{AST in extract or praziquantel}}}}{{\text{AST in Saline solution}}}} \right) \times 100$$

Toxicological endpoint for the parasites is death after observation under stereo-microscope by poking with a needle.

### Statistical analysis

The number of deaths on exposures at varying concentrations under the various treatments was analysed using regression (Probit analysis) – SPSS 11.0 version. The probit values were plotted against logarithm of concentration in order to determine the lethal concentration (LC_50_), which was metered to the varying concentrations; LC_5_, LC_10_ and LC_95_.

### Ethical approval and consent participate

This research was approved by the Faculty Research Ethics Committee (FREC), University of Lagos focused on the global protection, conservation, and sustainability of aquatic animals, fishery resources, and aquatic ecosystem. This approval also includes the consent to participate and consent to publish.

## Results

### Phytochemical analysis of plant extracts

#### Qualitative analysis

Tables 1 and 2 showed the qualitative phytochemical screening of the presence of alkaloids, tannin, phlobatannin, saponin, phenol, reducing sugar, steriods, cardaic glycoside, terpenoid and flavonoid in three different solvent extracts of five tropical plants. These three solvents include; 100% water, 100% ethanol and 70% diluted ethanol. All phytochemicals, except phlobatannin and reducing sugar were not present in 100% water and 100% ethanol extracts of neem and only phlobatannin was not present in 70% diluted ethanol. All phytochemicals, except phlobatannin and phenol were present in 70% diluted ethanol in extracts of moringa, tannin, phenol and phlobatannin were not present in 100% water extract while, alkaloid, phlobatannin phenol, steriods and cardiac glycoside were not detected in 100% ethanol extract. Phenol, phlobatannin, reducing sugar, steriods and cardiac glycoside were not detected in 100% water and 100% ethanol extracts of alligator pepper, but all phytochemicals were detected in 70% dilute ethanol extract. All phytochemicals were present in 70% diluted ethanol in extracts of garlic, tannin, phenol, phlobatannin and reducing sugar were not present in 100% water extract while, only reducing sugar was not detected in 100% ethanol extract. All phytochemicals were present in 70% diluted ethanol in extracts of ginger, phenol, phlobatannin, reducing sugar, steriods and cardaic glycoside were not present in 100% water extract while, only cardaic glycoside was not detected in 100% ethanol extract.

**Table 1:** Qualitative phytochemical parameters present in the extracts of neem, moringa and alligator pepper.

Phytochemicals	*Azadirachta indica* (Neem)			*Moringa cleifera* (Moringa)			*Afranamum melegueta* (Alligator pepper)		
	Water	Ethanol	W + Eth	Water	Ethanol	W + Eth	Water	Ethanol	W + Eth
Alkaloid	+	+	+ +	+	−	+	+ + +	+	+
Tannin	+	+	+ +	-	+ +	+ +	+	+ +	+
Phlobatannin	−	−	−	−	−	−	−	−	+
Saponin	+	+	+ + +	+ +	+	+ +	+ +	+ +	+ + +
Phenol	+	+	+	+ +	−	−	−	+	+
Reducing Sugar	−	−	+	−	+	+ +	−	−	+
Steriods	+	+	+ +	+ +	−	+	−	−	+
Cardiac Glycoside	+	+	+ +	+	−	+	−	−	+
Terpenoid	+ +	+ +	+ + +	+	+	+	+ + +	+	+ + +
Flavonoid	+ +	+	+	+ + +	+ +	+ +	+	+ +	+ + +

Signifies absence, + signifies mildly present, +  + signifies moderately present, +  +  + signifies highly present.

**Table 2:** Qualitative phytochemical parameters present in the extracts of garlic and ginger.

Phytochemicals	*Allium sativum* (Garlic)			*Zingiber officinale* (Ginger)		
	Water	Ethanol	W + Eth	Water	Ethanol	W + Eth
Alkaloid	+	+	+	+ +	+ + +	+ +
Tannin	−	+	+	+	+	+ + +
Phlobatannin	−	+	+	−	+	+
Saponin	+ + +	+	+ +	+ +	+	+ +
Phenol	−	+ +	+ +	−	+	+ +
Reducing Sugar	−	−	+	−	+	+
Steriods	+	+	+	−	+	+
Cardiac Glycoside	+ +	+	+ +	−	−	+
Terpenoid	+ +	+	+ +	+	+ +	+ + +
Flavonoid	+	+	+	+	+ +	+ +

Signifies absence, + signifies mildly present, +  + signifies moderately present, +  +  + signifies highly present.

#### Quantitation analysis

Tables 3 and 4 quantified the dominant phytochemical compounds present in the plant extracts. *Azadirachta indica* has alkaloids, tannin, flavonoid and saponin in ratios 1:1:4:3. It has more of flavonoid and saponin with mean concentrations; 1.84 ± 0.15 mg (100 g)^-1^ p < 0.05 and 1.32 ± 0.09 mg (100 g)^-1^ *p* < 0.05 respectively. *Moringa cleifera* has alkaloids, tannin, flavonoid and saponin in ratios 8:1:10:1 and has more of alkaloid and flavonoid with mean concentrations; 3.91 ± 0.45 mg (100 g)^-1^, p < 0.05 and 5.12 ± 2.75 mg (100 g)^-1^ respectively. Alkaloids, tannin, flavonoid and saponin are in ratios 1:1:3:9 in *Afranamum melegueta* with mean concentrations of flavonoid and saponin as 3.56 ± 1.89 mg (100 g)^-1^, and 9.94 ± 1.80 mg (100 g)^-1^, p < 0.05 respectively. *Allium sativum* and *Zingiber officinale* have alkaloids, tannin, flavonoid and saponin in ratios 6:2:1:4 and 6:3:2:1 respectively. *Allium sativum* has more of alkaloid and saponin with mean concentrations; 1.38 ± 0.22 mg (100 g)^-1^, *p* < 0.05 and 0.23 ± 0.11 mg (100 g)^-1^ respectively, while, *Zingiber officinale* has more of alkaloid and tannin with mean concentrations; 1.86 ± 0.07 mg (100 g)^-1^, p < 0.05 and 0.96 ± 0.05 mg (100 g)^-1^ *p* < 0.05 respectively.

**Table 3:** Quantitative phytochemical parameters present in the extracts.

Phytochemicals	Neem	Moringa	Alligator Pepper	Garlic	Ginger
Alkaloid, mg(100 g)^-1^	0.52 ± 0.12*	3.91 ± 0.45*	1.18 ± 0.03*	1.38 ± 0.22*	1.86 ± 0.07*
Tannin, mg(100 g)^-1^	0.45 ± 0.21	0.49 ± 0.03*	1.12 ± 0.05*	0.45 ± 0.04*	0.96 ± 0.05*
Flavonoid, mg(100 g)^-1^	1.84 ± 0.15*	5.12 ± 2.75	3.56 ± 1.89	0.94 ± 0.23	0.60 ± 0.22
Saponin, mg(100 g)^-1^	1.32 ± 0.09*	0.51 ± 0.31	9.94 ± 1.80*	0.23 ± 0.11	0.31 ± 0.23

Mean ± Standard Deviation; *Signifies M ± SD significant at *p* < 0.05.

**Table 4:** Ratios of the dominant phytochemical compounds present in the plant extracts.

Phytochemicals	Neem	Moringa	Alligator Pepper	Garlic	Ginger
Alkaloid, mg(100 g)^-1^	1	8	1	6	6
Tannin, mg(100 g)^-1^	1	1	1	2	3
Flavonoid, mg(100 g)^-1^	4	10	3	4	2
Saponin, mg(100 g)^-1^	3	1	9	1	1

#### *Lethal and Sub-lethal Concentrations of plant extracts and* praziquantel* on juvenile Fish*

Tables 5 and 6 show comparisons in the lethal and sub-lethal concentrations of five plant extracts and a chemo-anthelmintics on juvenile *C. gariepinus.* The lethal (96 h LC_50_, 96H LC_95_) and sub-lethal (96 h LC_5_, 96 h LC_10_) concentrations of five (5) plant extracts in comparison with the praziquantel on juvenile *C. gariepinus* shows that the praziquantel was more toxic than the plant extracts except for *Afranamum melegueta.* The toxicity of *Afranamum melegueta* on juvenile *C. gariepinus* (96 h LC_5_; 9.99 mg/l, 96 h LC_10_; 15.27 mg/l, 96 h LC_50_; 68.36 mg/l, 96 h LC_95_; 468.06 mg/l) was higher than Praziquantel (96 h LC_5_; 12.36 mg/l, 96 h LC_10_; 19.14 mg/l, 96 h LC_50_; 89.64 mg/l, 96 h LC_95_; 650.22 mg/l) with relative toxicity factors (RTFs) of *Afranamum melegueta* (11.0, 10.0, 5.0, 5.0), Praziquantel (9.0, 8.0, 4.0, 4.0) respectively.

**Table 5:** 96 Hour Acute toxicity of plant extracts and chemo-anthelmintics against juvenile of *Clarias gariepinus.*

Plant extracts	LC_5_ (mg/l)	LC_10_ (mg/l)	LC_50_ (mg/l)	LC_95_ (mg/l)	DF	SE	Confidence limit	Equation Y
*Azadirachta indica*	44.32	68.56	319.38	2301.0	4	0.48	0.98–2.86	1.69x + −4.2
*Moringa cleifera*	90.72	122.99	359.89	1428	4	0.64	1.49–4.00	2.57x + −6.5
*Afranamum melegueta*	9.99	15.27	68.36	468.06	4	0.51	0.96–2.98	1.67x + −3.0
*Allium sativum*	108.89	140.00	339.84	1061.0	4	0.74	1.88–4.77	3.13x + −7.75
*Zingiber officinale*	115.08	146.07	338.73	997.02	4	0.74	0.87–2.18	1.5x + −8.25
Albendazole	37.19	57.20	261.39	1837.4	4	0.21	0.44–1.25	0.83x + −4.41
Praziquantel	12.36	19.14	89.64	650.22	4	0.22	0.41–1.26	0.75x + −3.0

RTF – Relative Toxicity Factor, 96HrLC_5_- Lethal Concentration (5%), 96HrLC_10_- Lethal Concentration (10%), 96HrLC_50_- Lethal Concentration (5%), 96HrLC_5_- Lethal Concentration (95%),

**Table 6:** Relative toxicity factors (RTF) of plant extracts and chemo-anthelmintics at increasing toxicities against juvenile of *Clarias gariepinus*.

Plant extracts	LC_5_ (mg/l) (RTF)	LC_10_ (mg/l) (RTF)	LC_50_ (mg/l) (RTF)	LC_95_ (mg/l) (RTF)
*Azadirachta indica*	44.32 (3.0)	68.56 (2.0)	319.38 (1.0)	2301.0 (1.0)
*Moringa cleifera*	90.72 (1.0)	122.99 (1.0)	359.89 (1.0)	1428.0 (2.0)
*Afranamum melegueta*	9.99 (11.0)	15.27 (10.0)	68.36 (5.0)	468.06 (5.0)
*Allium sativum*	108.89 (1.0)	140.00 (1.0)	339.84 (1.0)	1061.0 (2.0)
*Zingiber officinale*	115.08 (1.0)	146.07 (1.0)	338.73 (1.0)	997.02 (2.0)
Albendazole	37.19 (3.0)	57.20 (2.0)	261.39 (1.0)	1837.4 (1.0)
Praziquantel	12.36 (9.0)	19.14 (8.0)	89.64 (4.0)	650.22 (4.0)

RTF – Relative Toxicity Factor, 96HrLC_5_- Lethal Concentration (5%), 96HrLC_10_- Lethal Concentration (10%), 96HrLC_50_- Lethal Concentration (50%), 96HrLC_5_- Lethal Concentration (95%),

Among the other four (4) extracts, *Azadirachta indica* was the most toxic. The toxicity of *Azadirachta indica* on juvenile *C. gariepinus* is 96 h LC_5_; 44.32 mg/l, 96 h LC_10_; 68.56 mg/l, 96 h LC_50_; 319.38 mg/l, 96 h LC_95_; 2301 mg/l with relative toxicity factors (RTFs) of 3.0, 2.0, 1.0 and 1.0. The order of increasing toxicity is shown as; *Azadirachta indica* < Praziquantel < *Afranamum melegueta.*

### *Low effect concentration (LOEC) of plant extracts and* chemo-anthelmintics* on post juvenile Fish*

Tables 7 and 8 show the behavioural responses of post juvenile of *Clarias gariepinus* exposed to lethal and sub-lethal concentrations of *Afranamum melegueta* and praziquantel. The lethal concentrations; 96 h LC_50_ and 96hL C_95_ of the plant extracts and the chemo-anthelmintics after 24 h of exposure showed rapid movement, faster opercular activity, erratic swimming, loss of balance, respiratory distress, lethargy and gulping for air. However, most of these behaviourial responses were not exhibited when exposed to sublethal concentrations; 96 h LC_5_ and96h LC_10_. Rather, at 96hLC_10_ concentration, the exposed fish showed rapid movement, faster opercular activity, erratic swimming. There were no significant observations seen in the exposed fishes to sublethal concentration, 96 h LC_5_ after 24 h, except in *Afranamum melegueta* with minimal response.

**Table 7:** Behavioural changes of Post juvenile of *Clarias gariepinus* Exposed to Various Concentrations of *Afranamum melegueta* at 24 h*.*

Plant extracts	Control	LC_5_ (mg/l)	LC_10_ (mg/l)	LC_50_ (mg/l)	LC_95_ (mg/l)
Rapid movement	−	+	+	+	+
Faster opercular activity	−	−	+	+	+
Erratic swimming	−	−	+	+	+
Loss of balance	−	−	−	+	+
Respiratory distress	−	−	−	+	+
Lethargy	−	−	−	+	+
Gulping for air	−	−	−	+	+

**Table 8:** Behavioral changes of Post juvenile of *Clarias gariepinus* Exposed to Various Concentrations of Praziquantel at 24 h.

Plant extracts	Control	LC_5_ (mg/l)	LC_10_ (mg/l)	LC_50_ (mg/l)	LC_95_ (mg/l)
Rapid movement	−	−	+	+	+
Faster opercular activity	−	−	+	+	+
Erratic swimming	−	−	+	+	+
Loss of balance	−	−	−	+	+
Respiratory distress	−	−	−	+	+
Lethargy	−	−	−	+	+
Gulping for air	−	−	−	+	+

### *Low Effect Concentration (LOEC) of plant extracts and* chemo-anthelmintics* on helminth parasites*

Table 9 shows the Average Survival Time (AST) of the intestinal helminths; *Wenyonia spp*, *Procamallanus spp, Tenuisentis spp* and *Electrotaenia spp* in 0.0085 mg/l Saline Solution, lethal (96 h LC_50_) and sub-lethal (96 h LC_5,_ 96 h LC_10_) concentrations of the five plant extracts and the two chemo-anthelmintics. *Wenyonia minuta,* a cestode and *Procamallanus longus* at 96 h LC_5_ and 96 h LC_10_, had the shortest survival time in *Afranamum melegueta* with percentage reduction in survival time relative to saline solution are 88.4% and 92.6%; 96.5% and 88.0% respectively. *Tenuisentis spp,* an acanthocephalan and *Electrotaenia malopteruri*, a cestode at 96 h LC_5_ and 96 h LC_10_, had the shortest survival time in *Afranamum melegueta* with percentage reduction in survival time relative to saline solution are 85.7% and 88.8%; 93.5% and 94.1% respectively.

**Table 9:** Average survival time (AST) and reduction in survival time (RST) of helminth parasites exposed to increasing toxicities of the extracts and chemo-anthelmintics.

Plant extracts	Parasites	LC_5_ AST (%RST)	LC_10_(%RST)	LC_50_ (%RST)
*Azadirachta indica* (Mean ± SD) minutes	*Wenyonia sp*	39.75 ± 4.27*(69.7%)	19.75 ± 1.50*(85.0%)	3.75 ± 0.96*(97.1%)
	*Procamallanus sp*	45.25 ± 6.40*(66.3%)	29.75 ± 2.22*(78.0%)	6.50 ± 1.3*(95.2%)
	*Tenuisentis sp*	35.50 ± 3.00*(66.8%)	17.50 ± 3.00*(83.6%)	2.75 ± 0.96(97.4%)
	*Electrotaenia sp*	25.50 ± 5.51*(79.3%)	12.75 ± 2.99*(89.7%)	1.50 ± 0.58(98.8%)
*Moringa cleifera* (Mean ± SD) minutes	*Wenyonia sp*	54.75 ± 7.5*(58.3%)	32.75 ± 4.6*(75.1%)	7.25 ± 2.4*(94.5%)
	*Procamallanus sp*	67.75 ± 5.7*(49.9%)	45.50 ± 4.0*(66.4%)	10.5 ± 17*(92.2%)
	*Tenuisentis sp*	47.25 ± 4.27*(55.8%)	33.75 ± 3.50*(68.4%)	6.0 ± 2.16(94.4%)
	*Electrotaenia sp*	34.25 ± 4.50*(72.2%)	20.50 ± 2.65*(83.4%)	4.75 ± 1.71(96.1%)
*Afranamum melegueta* (Mean ± SD) minutes	*Wenyonia sp*	15.25 ± 3.30*(88.4%)	9.75 ± 4.34(92.6%)	1.38 ± 0.48(98.9%)
	*Procamallanus sp*	21.75 ± 2.99*(86.5%)	16.25 ± 6.70(88.0%)	2.25 ± 0.50*(98.3%)
	*Tenuisentis sp*	15.25 ± 2.6*(85.7%)	12.00 ± 4.08*(88.8%)	0.63 ± 0.25(99.4%)
	*Electrotaenia sp*	8.00 ± 1.83*(93.5%)	7.25 ± 1.50*(94.1%)	0.87 ± 0.25(99.3%)
*Allium sativum* (Mean ± SD) minutes	*Wenyonia sp*	68.75 ± 6.2*(47.7%)	51.0 ± 6.59*(61.2%)	11.8 ± 2.4*(91.1%)
	*Procamallanus sp*	62.75 ± 4.6*(53.6%)	44.75 ± 5.4*(66.9%)	12.5 ± 2.9*(90.8%)
	*Tenuisentis sp*	51.0 ± 8.52*(52.3%)	36.00 ± 7.87*(66.3%)	8.75 ± 1.7*(91.8%)
	*Electrotaenia sp*	36.0 ± 8.29*(70.8%)	23.50 ± 8.99(80.9%)	6.0 ± 1.63*(95.1%)
*Zingiber officinale* (Mean ± SD) minutes	*Wenyonia sp*	62.25 ± 6.1*(52.6%)	42.3 ± 4.79*(67.8%)	11.7 ± 4.6(91.1%)
	*Procamallanus sp*	54.50 ± 5.80*(59.7%)	34.75 ± 6.90*(74.3%)	11.0 ± 2.9*(91.9%)
	*Tenuisentis sp*	45.75 ± 6.18*(57.2%)	32.75 ± 6.34*(69.4%)	9.5 ± 1.29*(91.1%)
	*Electrotaenia sp*	33.75 ± 6.75*(72.6%)	23.50 ± 4.20*(80.9%)	4.5 ± 1.29*^(^96.4%)
Praziquantel (Mean ± SD) minutes	*Wenyonia sp*	18.75 ± 2.1*(85.7%)	13.11 ± 7.1(90.0%)	2.1 ± 0.09*(98.4%)
	*Procamallanus sp*	25.26 ± 9.2(81.3%)	21.45 ± 2.9*(84.1%)	3.52 ± 0.7*(97.4%)
	*Tenuisentis sp*	23.50 ± 4.2*(78.0%)	19.32 ± 1.8*(81.9%)	4.15 ± 1.6*(96.1%)
	*Electrotaenia sp*	11.25 ± 1.7*(90.9%)	8.45 ± 4.8(93.1%)	1.25 ± 0.7(99.0%)

Mean ± Standard Deviation; *Signifies M ± SD significant at p < 0.05; AST – Average Survival Time; %RST – Percentage Survival Time; 0.0085 mg/l Saline Solution (in minutes); *Wenyonia sp* (131.33 ± 7.09*), *Procamallanus sp* (135.33 ± 10.02*), *Tenuisentis sp* (106.87 ± 16.07*), *Electrotaenia sp* (123.33 ± 10.41*).

**Figure 1:**
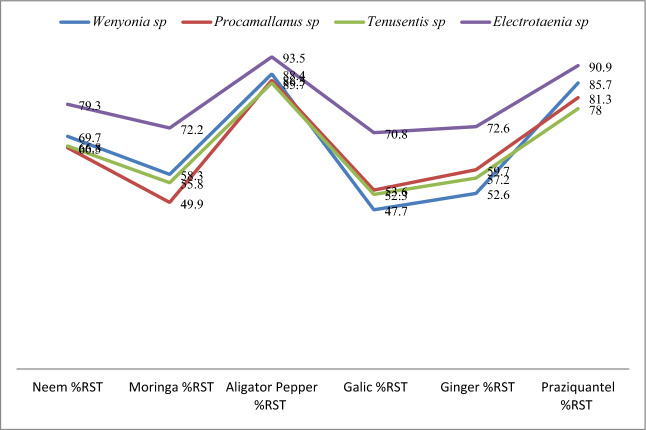
Percentage Reduction in Survival Time (%RST) of Helminth parasites Exposed to the Extracts and chemo-anthelmintics at 96 Hour LC_5_ of fish juvenile*.*

**Figure 2:**
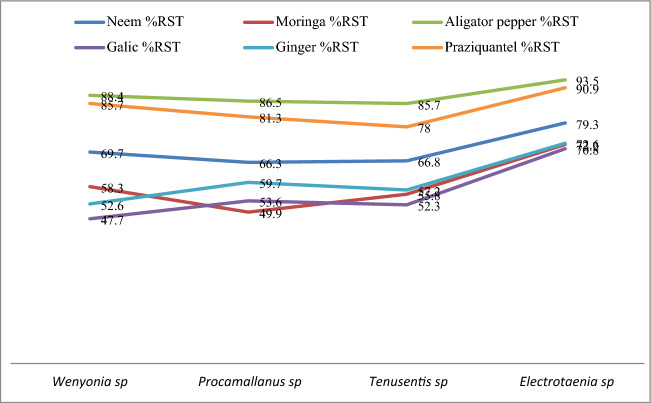
Comparison of Potency of the Plant Extracts and chemo-anthelmintics against Helminth parasites at 96 Hour LC_5_ of fish juvenile*.*

However, *Afranamum melegueta* and Praziquantel showed above 70% potency (at 96 h LC_5_) at against all parasites. *Azadirachta indica, Moringa cleifera, Allium sativum* and *Zingiber officinale* showed above 70% potency (at 96 h LC_5_) against *Electrotaenia malopteruri* as shown in Figs. 1 and 2. The order of increasing toxicity of the plant extracts and the chemo-anthelmintics on the parasites is shown as; *Azadirachta indica* < Praziquantel < *Afranamum melegueta. Electrotaenia malopteruri* was the most susceptible among the parasites to the plant extracts and the chemo-anthelmintics. The order of decreasing susceptibility to *Afranamum melegueta* and Praziquantel is shown as; *Electrotaenia sp* < *Wenyonia spp* < *Procamallanus spp* < *Tenuisentis spp..* However, *Moringa cleifera* and *Azadirachta indica* showed a different trend, *Electrotaenia spp* < *Wenyonia spp* < *Tenuisentis spp* < *Procamallanus spp,* while, *Allium sativum* and *Zingiber officinale* had *Electrotaenia spp* < *Procamallanus spp* < *Tenuisentis spp* < *Wenyonia spp.*

## Discussion

Plants are rich in chemical compounds that might serve as alternatives to the use of chemotherapeutants. In this study, the qualitative and quantitative phytochemical screening of five tropical plant species; *Azadirachta indica* (neem), *Moringa cleifera* (moringa), *Afranamum melegueta* (alligator pepper), *Allium sativum* (garlic) and *Zingiber officinale* (ginger) in different solvents (100% ethanol, 100% water and 70% ethanol)* i*ndicate the presence of these phytochemicals in different proportions. The results obtained show that the 70% ethanol extract had 80% to 100% presence of the phytochemical content, compared with 100% aqueous and 100% ethanol extracts with 50% to 80% and 50% to 90%, respectively. This could be because the phytochemical contents have different polarities. Some of them are better extracted in aqueous phase, while some in the organic phase, using a correct proportion of aqueous-organic phase rather than an organic phase or the aqueous phase alone which agrees with the findings of Kala et al.,^18^, Singh and Chauhan,^19^ and^20^,

The present study shows the presence of alkaloids, tannin, flavonoid and saponin as dominant phytochemical compounds across the five plant species. Previous studies reported isolated steroidal saponins from the rhizome of *Paris polyphylla* (ginseng), and five alkaloids from *Macleaya microcarp a*nd flavonoids from the rhizome of *D. rhamnoside*s had strong efficacy against monogenean *D. intermedius*^21–23^* .* Among the five tropical plants, the richest in saponin and flavonoids are alligator pepper and neem with alkaloids, tannin, flavonoid and saponin in ratios 1:1:3:9 and 1:1:4:3 respectively. While, moringa, garlic and ginger are rich in alkaloids with alkaloids, tannin, flavonoid and saponin in ratios, 8:1:10:1, 6:2:1:4 and 6:3:2:1 respectively.

Alternatively, natural products are preferred to conventional chemical treatment against parasites in aquaculture due to the their biodegradability, environmental safety, non-bio accumulation and mitigating parasite resistance^24–26^, The present study showed comparisons in the lethal and sub-lethal concentrations of the five plant extracts and chemo-anthelmintics on different classes of helminth parasites and fish host using *Clarias gariepinus* as a fish model. *Afranamum melegueta* (alligator pepper) is the most toxic of the five plant extracts and more toxic than the chemo-anthelmintics (praziquantel). Praziquantel is a widely used anthelmintic, safe and effective bath treatment with wide margin of safety for the removal of a range of parasites of fish^27^, ^28^; ^29,30^,

*Afranamum melegueta* and Praziquantel showed above 70% potency (at 96 h LC_5_) against all the classes of parasites; *Wenyonia spp* (cestode), *Procamallanus spp* (nematode),*, Tenuisentis spp* (acanthocephalane), and *Electrotaenia sp* (cestode) as compared to the other plant extracts that showed above 70% potency (at 96 h LC_5_) only against *Electrotaenia spp* (see Figs. 1 and 2). *Electrotaenia spp,* a proteocephalidean cestode is the most susceptible of the parasites, Sublethal Concentrations96 hr LC_5_ of praziquantel and A*franamum melegueta* on the juvenile fish host (12.36 mg/l and 9.9 mg/l respectively) were found to be 90,9% and 93,5% effective against adult *Electrotaenia spp* after 8 to 10 min of exposure. These concentrations were 78%to 85,7% and 89.7% to 88,4% respectively, effective against the other classes of parasites after 18 to 25 min and 15 to 21 min of exposure. These concentrations were tested on the post juvenile of the fish to determine behavioral changes, there were no significant behavioral responses after 24 h of exposure (see Tables 7 and 8). The effective concentrations indicate a wide margin of safety for the fish host. This study is in agreement with the findings of Vaughan and Chisholm, (2010), Hadfield and Clayton,^29^ for praziquantel and Akinsanya et al.,^9^ and Akinsanya et al.,^10^ for A*franamum melegueta.*

## Conclusion

In summary, this study demonstrated the potential of A*franamum melegueta* with other plant extracts as alternative anthelmintics in the treatment of helminth parasite infection in fishes. A*franamum melegueta* showed high potency with the widest margin of safety for the fish host.

## Data Availability

Data and materials are available at the Department of Zoology Archive, University of Lagos.
